# Tacrolimus Induces Insulin Resistance and Increases the Glucose Absorption in the Jejunum: A Potential Mechanism of the Diabetogenic Effects

**DOI:** 10.1371/journal.pone.0143405

**Published:** 2015-11-23

**Authors:** Zhiwei Li, Fei Sun, Yaohui Zhang, Hao Chen, Ningning He, Hui Chen, Penghong Song, Yan Wang, Sheng Yan, Shusen Zheng

**Affiliations:** 1 Division of Hepatobiliary and Pancreatic Surgery, Department of Surgery, First Affiliated Hospital, School of Medicine, Zhejiang University, Hangzhou, China; 2 Collaborative Innovation Center for Diagnosis and Treatment of Infectious Diseases, Hangzhou, China; Baylor College of Medicine, UNITED STATES

## Abstract

**Background:**

The use of the immunosuppressive drug tacrolimus (TAC) is related to new onset diabetes after transplantation. Herein, we examined the effect of intraperitoneal administered TAC on intestinal glucose absorption in mice.

**Methods:**

Animals received low, medium, or high dose TAC (0.5, 1, or 5 mg/kg/d, respectively), or 0.9% saline solution (control) for 14 days. Oral glucose tolerance test (OGTT), insulin concentration test, and serum TAC concentration measurements was performed after 14 days of TAC exposure. Plasma insulin was assessed and electrogenic glucose absorption were measured by the sodium-dependent increase of the short-circuit current. Expression levels of the glucose transporters sodium glucose co-transporter (SGLT) 1, glucose transporter (GLUT) 2, and GLUT5 were also determined.

**Results:**

Oral glucose absorption assessed by OGTT was significantly enhanced in the low, medium, and high groups. Serum insulin was elevated in the medium and high group compared with the control. Moreover, glucose-induced Isc was significantly higher in TAC administrated groups, which indicates that SGLT1 activity increased. Transcription levels and protein abundance of SGLT1 in the experimental groups also increased compared with the control.

**Conclusions:**

TAC induced insulin resistance and strengthened intestinal glucose absorption by increasing the activity and expression of the glucose transporter, SGLT1.

## Introduction

New-onset diabetes after transplantation (NODAT) is a severe complication following organ transplantation and has been reported to occur in between 2% and 53% of transplanted patients [1]. Post-transplant hyperglycemia is related to risks of infection, cardiovascular disease [2], and graft dysfunction [3], and is associated with higher mortality [4]. Although there are a multitude of factors that contribute to the development of NODAT, the dominant factor is the use of immunosuppressive agents, such as corticosteroids, calcineurin-inhibitors (CNIs), and sirolimus, amongst others [5]. Tacrolimus (TAC) and cyclosporine are two widely used calcineurin-inhibitor drugs and both have diabetogenic effects [6]. The use of TAC is associated with an incidence of NODAT that is 2.5-fold greater than cyclosporine [1, 7, 8].

The mechanism underling the diabetogenic effect of TAC is currently being investigated [6, 9]. There are many studies focusing on changes in β-cell regeneration, insulin secretion, and insulin resistance after TAC administration [10–12]. Although glucose absorption in the intestines plays a crucial role in glucose homeostasis [13], it is unknown if intestinal glucose absorption is involved in the diabetogenic effect of TAC. According to a previous study, glucose malabsorption in the jejunum after TAC treatment was observed and the effect was dose-dependent [14]. Therefore, it is reasonable to hypothesize that intestinal glucose absorption participates in the diabetogenic TAC process. Thus, the aim of this study was to study the influence of different doses of TAC on glucose absorption in the intestine and to evaluate glucose homeostasis.

In the small intestine, glucose is mainly transported by sodium glucose co-transporter 1 (SGLT1), glucose transporter 2 (GLUT2), and GLUT5 [15]. SGLT1 transports glucose by coupling with Na^+^ and generates a short-circuit current (Isc) that can be measured by using an Ussing chamber [16]. Thus, to some extent, Isc represents the activity glucose transport by SGLT1. GLUT2 and GLUT5 are Na +-independent transporters that transport monosaccharides and fructose, respectively, from the intestinal lumen into enterocytes and are dependent on a concentration gradient [15]. Of these transporters, SGLT1 is the major route for the transport of sugars from the lumen into enterocytes. Therefore, we investigated changes in activity and expression of these glucose transporters, mainly focusing on SGLT1, to elucidate the pathophysiological mechanism TAC on intestinal glucose absorption.

## Materials and Methods

### Mice

Male, 8-week-old C57BL/6 mice (Shanghai Animal Center, Chinese Academy of Science, Shanghai, People’s Republic of China) weighing 22–25 g were used for the experiments. The mice were housed under a strict 12:12 hour light-dark cycles (lights on at 7 AM) at 20°C in standard pathogen-free conditions and allowed unrestricted access to standard rodent chow (Zhejiang Academy of Medical Sciences) and water. Every mouse was kept in a plastic box called an Individually Ventilated Cage (IVC) measuring 325 mm x 210 mm x 180 mm.

Animal in vivo procedures in this study were performed according to the guidelines of the Institutional Animal Care and Use Committee (IACUC) at Zhejiang University, and the ethics committee of Zhejiang University also approved this study.

### Groups and tissue harvest

A total of 24 C57BL/6 mice were used in the present study. By the completely random design, all mice were allocated into four groups according to TAC dose: 0.5 mg (low dose group), 1 mg (medium dose group), and 5 mg/kg (high dose group) for experimental groups, and 0.9% saline solution for the control group. Each group consisted of six mice.

TAC was supplied by Astellas Pharma, Inc. (Tokyo, Japan) and dissolved in 0.9% saline solution. Equivalent volumes of TAC and saline were administered intraperitoneally (i.p.) to the mice each morning for 14 days.

To minimize the influence of diurnal variety of glucose transporters [17], tissues from each group were harvested at 10 am. Mice were humanely euthanized via overdose with an intraperitoneal injection of sodium pentobarbitone anesthetic, according to standardized protocols supplied by the Zhejiang University’s Ethics Committee 14 days after continuous intraperitoneal injection. All procedures were performed at Zhejiang University’s Animal Experimentation Facility laboratories. After animals were anesthetized and sacrificed, blood samples were immediately collected through cardiac puncture and centrifuged at 3000 rpm for 15 minutes, and then the plasma was removed and frozen at −80°C until they were assayed.

Blood TAC levels were measured by immunoassay on an IMx analyzer (Abbott Diagnostics Laboratories, Abbott-Park, IL, USA) [18]. Since the study focused on the glucose absorption in the intestine, 4 cm jejunum segments at about 4 cm distal to the ligament of Treitz were collected. Next, 2 cm of this segment were used for Ussing chamber experiments. The remnant tissue was divided into two parts, one of which was snap frozen in liquid nitrogen and stored at –80°C, while the other was fixed in 10% formaldehyde for 24 hours, dehydrated, and embedded in paraffin.

### Oral glucose tolerance test (OGTT) and insulin concentration tests

After fasting for 12 hours (9 pm to 9 am) with free access to water, mice were gavaged with D-glucose (3 g/kg body weight) in 0.9% saline solution. Blood samples were obtained from the tail vein before and after 20, 40, 60, and 80 minutes of glucose administration. Blood glucose was measured with a glucometer. Plasma insulin content was measured following glucose administration. At the above-mentioned time points, 100 μl of blood was collected [19] and plasma was isolated by low-speed centrifugation (2000 rpm, 5 minutes, 4°C). Plasma insulin was assessed by enzyme-linked immunosorbent assay (ELISA; Millipore, Watford, UK).

### Histopathology

Intestinal morphology was assessed using our previously published method [16]. Briefly, 6-μm sections were made from tissues embedded in paraffin and stained with hematoxylin and eosin (H&E). The height and width of villus and crypt depth were measured in at least three animals and segments in each group. Villus surface was evaluated as π*(villus length * villus width) [20].

### Electrophysiology

The jejunum segments were collected and incubated in ice-cold, gassed KRB solution containing 1 μM indomethacin for 10 minutes as previously described [21]. Briefly, the intestine was split longitudinally, and the seromusculature layers were removed and clamped into an Ussing chamber with an opening of 0.625 cm^2^. Under control conditions, the serosal and luminal perfusate contained (in mM): 105 NaCl, 2 KCl, 1 MgCl2, 1.25 CaCl2, 0.4 KH2PO4, 1.6 K2HPO4, 5 Na pyruvate, 25 NaHCO3, and 20 mannitol (pH 7.4, NaOH) [16]. The solutions were gassed with 95% O_2_ and 5% CO_2_ 1 hour before the Ussing chamber experiment. The chamber temperature was maintained by a circulating water bath at 37°C. Next, the empty chamber was placed into the apparatus and the “potential difference” across the empty chamber set to “0 mV” before mounting the tissue into the Ussing chamber [22]. The basal level of the transepithelial potential difference (Vt) and the short-circuit current (Isc) was recorded continuously for 30 minutes, then 20 mM mannitol were replaced by 20 mM glucose in the luminal perfusate, and the recording continued for another 60 minutes. A further single jejunum segment from each group mice was harvested using the same preprocessing as above. The basal level of the transepithelial potential difference (Vt) and the short-circuit current (Isc) was recorded continuously for 30 minutes, then 20 mM mannitol were replaced by 20 mM glucose in the luminal perfusate, and the recording continued for another 30 minutes. Finally, 20 mM glucose were replaced by another 20 mM glucose solution simultaneously containing 100 μM/L LX-4211 (Lexicon pharmaceuticals) in the luminal perfusate, and the recording continued for another 60 minutes. The mean value was taken for final analysis. The results were expressed as the intensity of the Isc (μA/cm^2^) after glucose challenge over basal Isc.

### Quantitative polymerase chain reaction

According to the methods of our previous study [16], total RNA was extracted from intestinal tissue using TRIzol reagent (Invitrogen, Carlsbad, CA, USA). cDNA was reverse transcribed from total RNA (2 μg) using M-MLV Reverse Transcriptase (Promega, San Luis Obispo, CA, USA). Quantitative real-time PCR (qRT-PCR) was performed with an ABIPRISM 7500 Sequence Detection System (Applied Biosystems) using the SYBR Premix Dimer Eraser kit (Takara Biotechnology, Dalian, Liaoning, People’s Republic of China). With a total volume of 10 μl, amplification reactions, which included 1 μl cDNA template, 0.3 μl each of forward and reverse primers (10 μM), 0.2 μl of 50X ROX Reference Dye II (Takara), and 5 μl of 2X SYBR Premix Dimer Eraser. The primers were used as per our previous study [16]: sglt1 5ʹ-CAGTAACATTGGAAGTGGTCA-3ʹ (forward) and 5ʹ-GGGACAGAACGGAAAGGT-3ʹ (reverse), glut2 5ʹ-TTGCTGGACGAAGTGTATC-3ʹ (forward) and 5ʹ-GACTAATAAGAATGCCTGTGAC-3ʹ (reverse), glut5 5ʹ- CCACTGTCCATTGCTACC-3ʹ (forward) and 5ʹ-TTAGACATGCTCCTTTGATT-3ʹ (reverse), GAPDH 5ʹ-GGTGAAGGTCGGTGTGAACG-3ʹ (forward), and 5ʹ-CTCGCTCCTGGAAGATGGTG-3ʹ (reverse). Amplification of the transcripts involved an initial denaturation at 95°C for 30 s, followed by 40 cycles at 95°C for 5 s, 55 C for 30 s, and 72°C for 34 s, and melting curve analysis was added after the final PCR cycle. The relative RNA expression was calculated using the delta-delta threshold cycle (ΔΔCT) method and normalized to GAPDH expression in the same cDNA sample.

### Western blotting

Using our previously published method, proteins were extracted and concentrations determined [16]. Next, 20 μg of protein were separated by sodium dodecyl sulfate polyacrylamide gel electrophoresis (SDS-PAGE) and transferred onto polyvinylidene fluoride (PVDF) membranes. After blocking nonspecific binding sites for 2 hours in TBST (1X TBS, 0.1% Tween 20) containing 4% non-fat milk and 1% bovine serum albumin (BSA), the membranes were incubated overnight on ice with the primary antibodies against SGLT1 (EMD Millipore Corporation, Billerica, MA, USA; 1:3000), GLUT2 (EMD Millipore Corporation; 1:3000), GLUT5 (EMD Millipore Corporation; 1:3000) and β-actin (Sigma-Aldrich; 1:3000). After washing three times in TBST, the membranes were incubated for 1 hour with horseradish peroxidase-conjugated goat anti-rabbit secondary antibodies, and then visualized with enhanced chemiluminescence detection kit (Biological Industries, Beit Haemek, Israel) and intensities were quantified using Image-Pro plus 6.0 software (Media Cybernetics, Bethesda, MD, USA).

### Statistical analysis

Data was analyzed using GraphPad-Prism 6 software (GraphPad-Prism Software Inc., San Diego, CA, USA) and provided as means ± standard error of the mean (SEM), where n represents the number of independent experiments. Differences between experimental groups and controls were assessed by Student *t*-test with or without Welch correction where applicable, and *P* < 0.05 was considered statistically significant.

## Results

### Body weight and food intake

**Fig 1:**
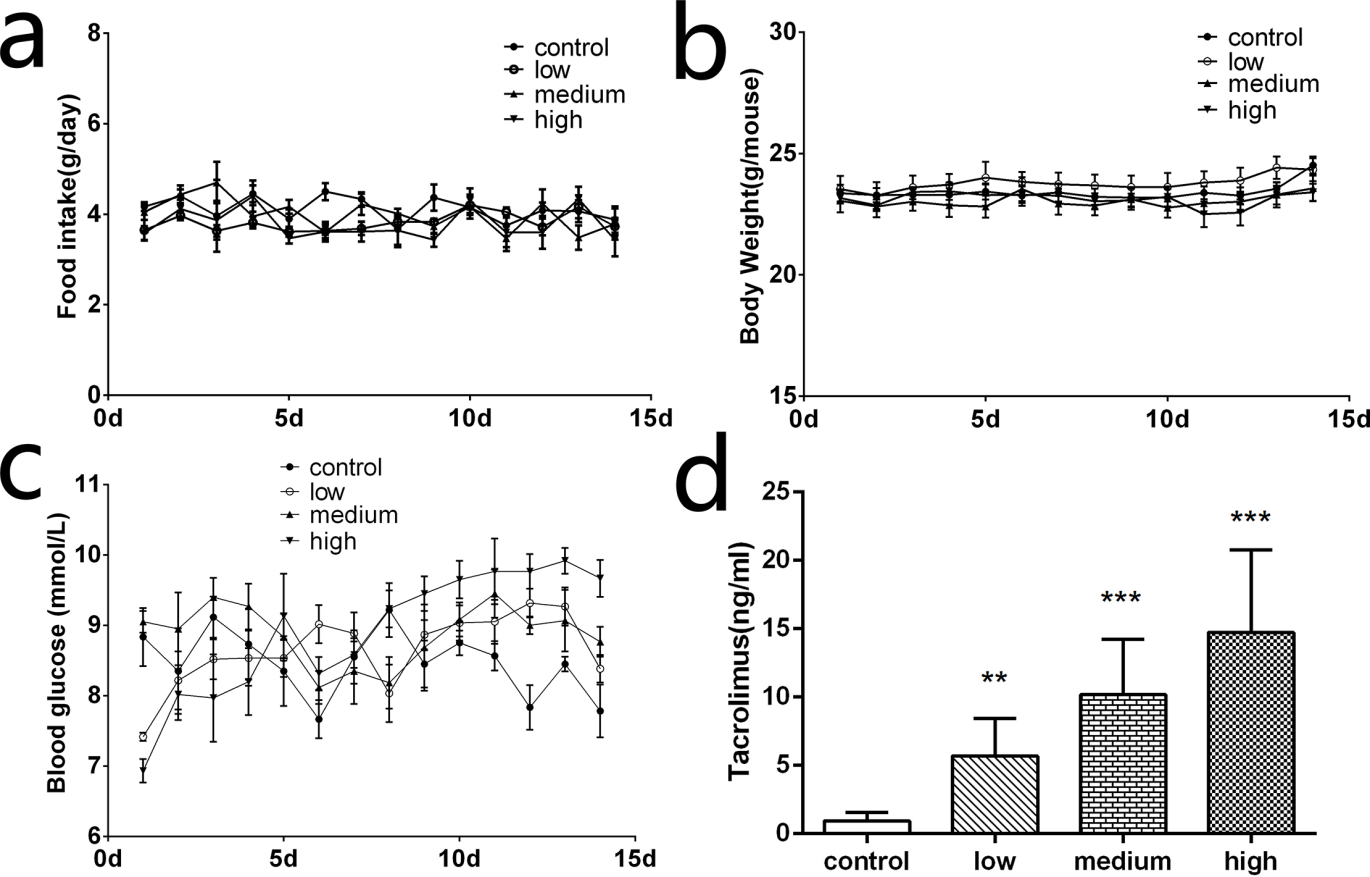
Body weight, food intake, random blood glucose, and blood TAC concentration. Body weight (a) and food intake (b) did not differ between groups in 14 days. (c) Random blood glucose was measured during the 14 experiment days. The use of TAC seemed to increase the blood glucose level and this effect was dose dependent. (d) Blood TAC concentration was dependent on TAC dose administrated in every group (*n* = 6, **P < 0.01, ***P < .001).

As the occurrence of clinical diabetes is often accompanied by changes in diet and weight, we monitored the food intake and body weight of each mice daily for 2 weeks. Surprisingly, food intake and body weight of mice during the observation remain stable and no significant difference was found between the four groups (Fig 1A and 1B).

### Random blood glucose

Random blood glucose of each mouse was monitored daily during the research period (Fig 1C). The control group maintained stable blood glucose levels. During 2 weeks of TAC administration, random blood glucose from the low and medium group rose slightly before the beginning of the experiment. In the high dose group, random blood glucose was obviously higher than basal levels, and also appeared higher level compared with the other three groups.

### TAC blood concentration

After injecting TAC daily for 14 days, blood samples of each mouse were collected and blood TAC concentrations were measured. Blood concentration of TAC in low, medium, and high groups were compared with the control group, as shown in Fig 1D: low: 5.692 ± 1.219 ng/mL, *P* = 0.0052; medium: 10.18 ± 1.811 ng/mL, P = 0.001; high: 14.73 ± 2.705 ng/mL, *P* = 0.001; control: 0.9400 ± 0.2766 ng/mL. Among the groups, blood TAC concentration in the low and medium group were within the effective therapeutic concentration and equivalent to those seen in human transplant patients [23].

### Oral glucose tolerance test (OGTT)

**Fig 2:**
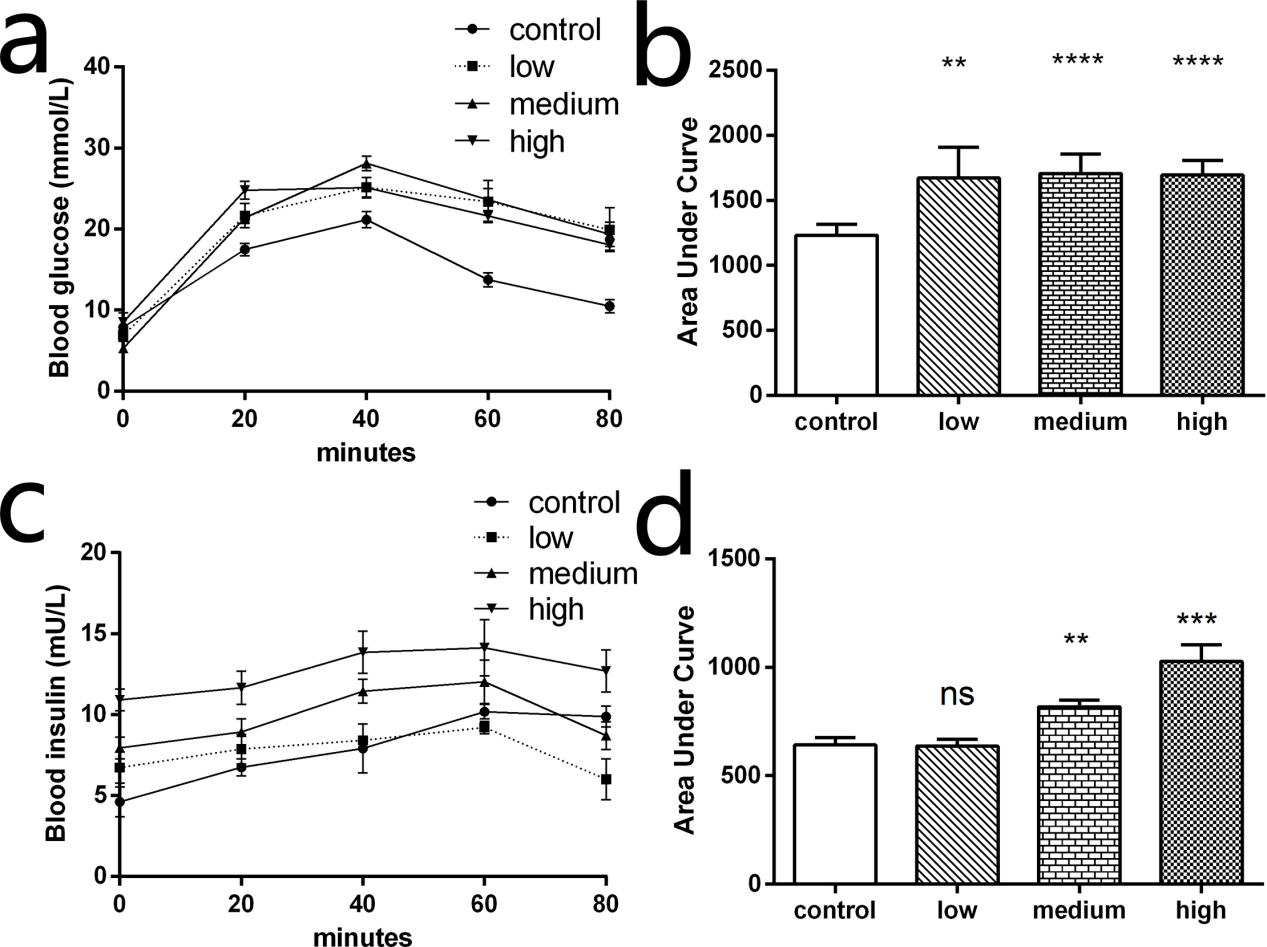
Oral glucose tolerance test (OGTT) and insulin concentration test. An OGTT (3 g/kg) was performed after drug administration for 14 days. (a) Blood glucose in TAC treated mice was significantly higher than those treated with saline during the test, indicating that glucose absorption increased after TAC administration. (b) The area under the curve for the low, medium and high group were also higher than control group. (c) Plasma insulin content was measured in mice following oral glucose administration. The medium and high dose groups displayed lowered glucose-stimulated insulin secretion. (d) The area under the curve for insulin releasing test show the medium and high dose groups were higher than the control group (*n* = 6, **P < 0.01, ***P < 0.001, ****P < 0.0001; ns indicates no significant difference).

OGTT was performed to evaluate changes in glucose metabolism after TAC administration. As was shown in Fig 2A, the blood glucose levels of low, medium, and high groups were significantly higher than the control group at each time points during OGTT. Compared with the control group, the area under curve was significantly increased in low, medium, and high groups (*P* < 0.001; Fig 2B). This implies that TAC increased the absorption of glucose in intestines or reduced glucose metabolism.

Using the insulin concentration test, we found increased levels of plasma insulin in the high and medium group compared with the control group (Fig 2C). Moreover, the area under curve was significantly higher in the medium (*P* = 0.0037) and high (*P* = 0.0008) groups compared to the control group (Fig 2D). No obvious difference was found between the low and control groups.

### Electrogenic Glucose Transport

**Fig 3:**
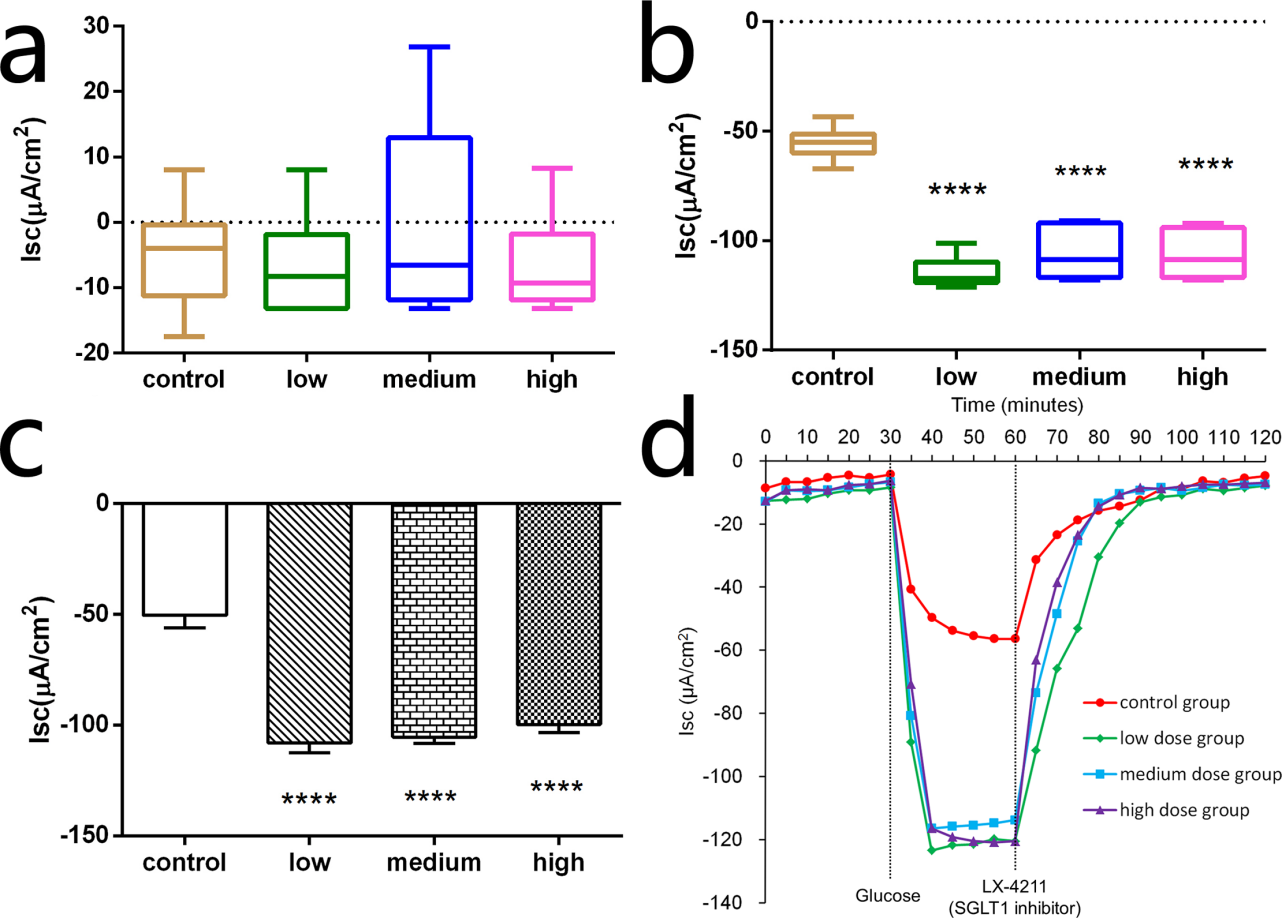
Electrogenic glucose transport. (a) Basal Isc was not significantly different between groups. (b) The addition of 20 mM glucose on the luminal side of the Ussing chamber significantly decreased the negative Isc, which was significantly lower in the low, medium, and high dose groups than the control group. (c) The addition of 20 mM glucose induced a sharp decrease in Isc (ΔIsc). ΔIsc was significantly increased in the low, medium, and high dose groups compared with the control group (*n* = 6, ****P < 0.0001). (d) The assay repeated in the presence of 100 μM/L LX-4211, confirming glucose transport measured by the Ussing chamber represents SGLT1.

Electrophysiological analysis was used to evaluate changes in intestine glucose transportation after TAC administration. In the absence of glucose, basal Isc was not significantly different from the control group (–4.95 ± 3.40 μA/cm^2^), low group (–6.67 ± 3.30 μA/cm^2^), medium group (–0.43 ± 6.28 μA/cm^2^) or high group (–6.67 ± 3.20 μA/cm^2^) at any time point (Fig 3A); this revealed that the basal activity of SGLT1 among the four groups were equivalent. A rapid and marked lumen-negative increase of Isc was induced after partial iso-osmotic replacement of 20 mM mannitol by 20 mM glucose in the luminal side (Fig 3B). The decrease in Isc induced by glucose in low (–114.6 ± 2.93 μA/cm^2^, *P* < 0.0001), medium (–105.8 ± 4.89 μA/cm^2^, *P* < 0.0001), and high groups (–106.4 ± 4.54 μA/cm^2^, *P* < 0.0001) were significantly higher than the control group (–55.36 ± 3.12 μA/cm^2^). Delta Isc (ΔIsc) represented the decrease in Isc after glucose presentation and indicates the enhanced activity of SGLT1. As shown in Fig 3C, ΔIsc in low (–107.9 ± 4.56 μA/cm^2^, *P* < 0.0001), medium (–105.4 ± 2.97 μA/cm^2^, *P* < 0.0001) and high groups (–99.75 ± 3.52 μA/cm^2^, *P* < 0.0001) were significantly higher than in control groups (–50.41 ± 5.74 μA/cm^2^). Glucose transport was completely inhibited when 100 μM/L LX-4211 was added to the glucose solution (Fig 3D), suggesting dependence on SGLT1. These findings indicate that electrogenic glucose transport in the jejunum significantly increased after administration of TAC. In other words, TAC enhanced intestine glucose transport through SGLT1.

### Histology

**Fig 4:**
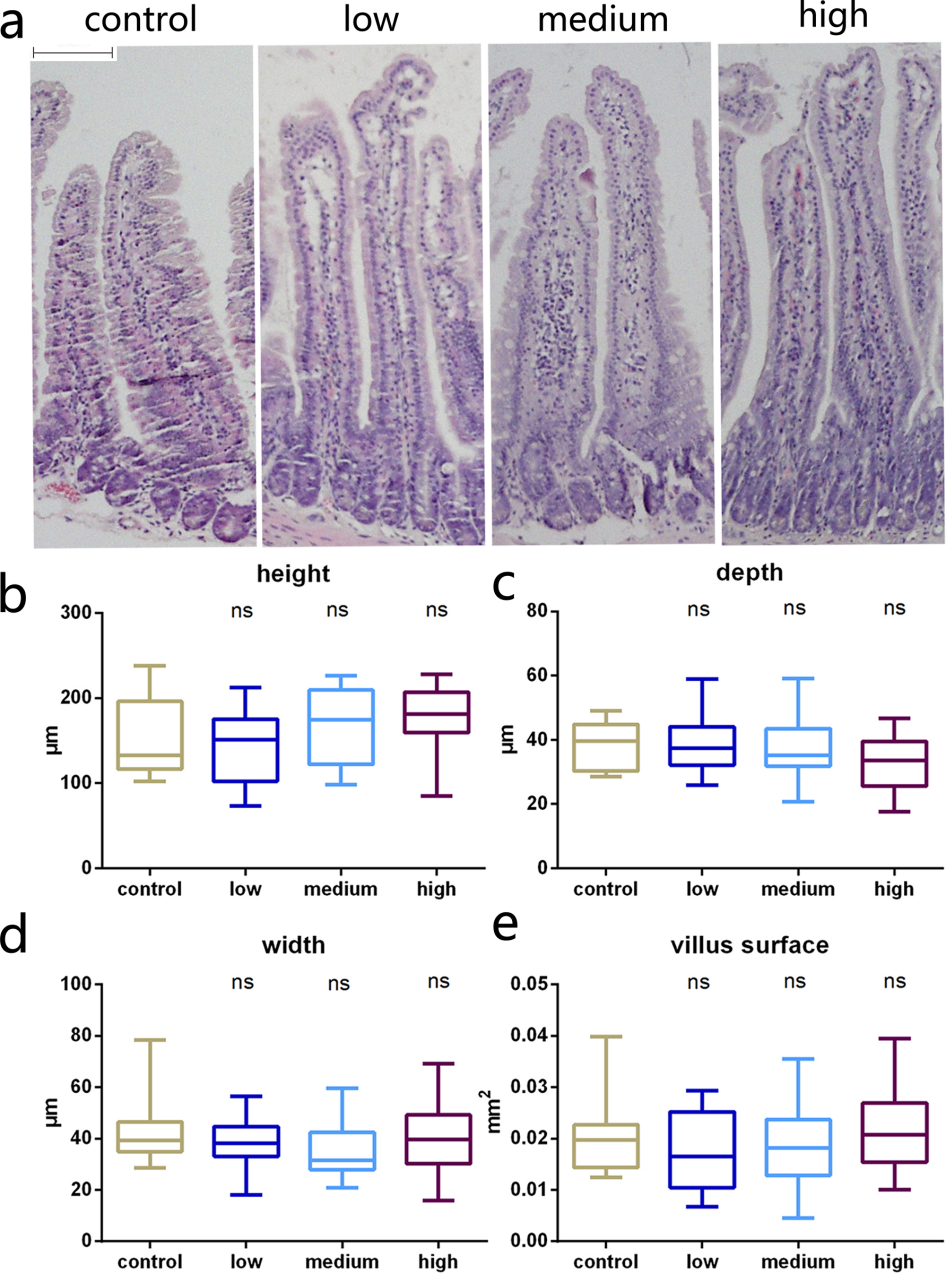
Intestinal histology. (a) Micrographs of representative sections from the four groups of mice (control, low, medium, and high dose; hematoxylin and eosin, ×100). Histological comparisons of jejunal segments from the four groups show villus height (b), villus width (c), crypt depth (d), and villus surface (e) did not differ after receiving different TAC doses (*n* = 6, ns indicates no significant difference). Scale bar: 50 μm.

With respect to villi height and width, crypt depth, and the villus surface, no significant differences were observed between the four groups (Fig 4). This result suggests that TAC may have no effect on intestine morphology.

### Transporter transcription and expression

**Fig 5:**
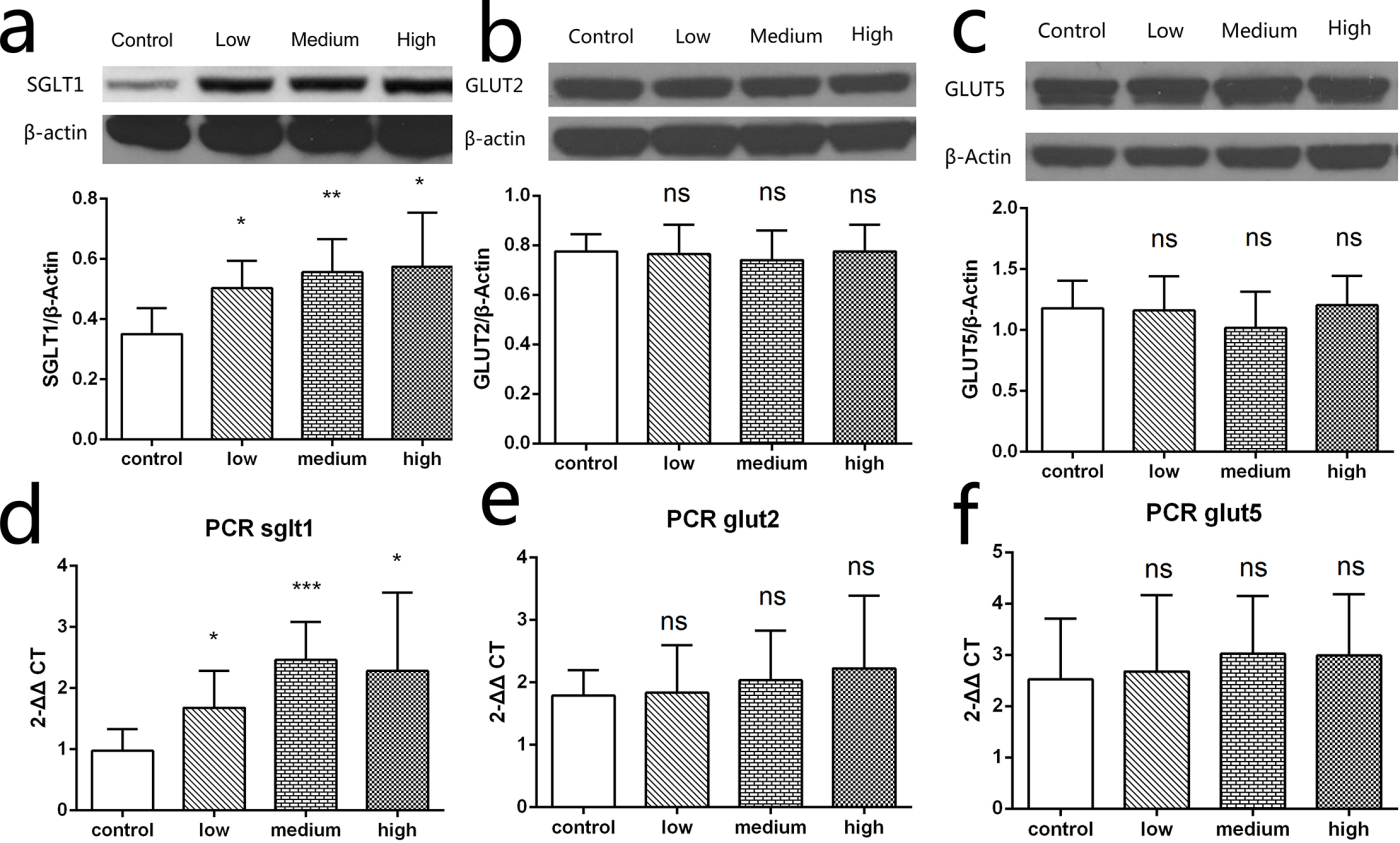
Transcription and expression of SGLT1, GLUT2, and GLUT5. (d) Transcription of sglt1 was increased in low, medium, and high dose groups compared with the control group. Transcription of glut2 (e) and glut5 (f) did not changed after TAC administration. Protein abundance of SGLT1 was determined by Western blot normalized to β-actin. (a) SGLT1 expression in low, medium, and high dose groups were significantly higher than the control group. For GLUT2 (b) and GLUT5 (c) expression, no significant difference was observed between groups (*n* = 6, *P < 0.05, **P < 0.01, ***P < 0.001, ns indicates no significant difference).

We further investigated gene transcription and protein expression levels of intestine glucose transporters, including SGLT1, GLUT2, and GLUT5. Consistent with the electrophysiology results, the relative transcription of sglt1 mRNA in low, medium, and high groups rose by 72% (*P* = 0.0339), 152% (*P* = 0.0005) and 134% (*P* = 0.037), respectively compared with the control group (Fig 5D). In accordance with mRNA results, the low, medium, and high groups showed significantly increased SGLT1 protein abundance compared with the control group (*P* = 0.013, *P* = 0.0047, and *P* = 0.0197, respectively; Fig 5A). However, both gene transcription levels and protein expression levels of GLUT2 (Fig 5B and 5E) and GLUT5 (Fig 5C and 5F) in the low, medium, or high groups were not significantly different from controls.

## Discussion

In the current study, we have found impaired glucose tolerance and increased plasma insulin levels in mice after TAC administration. Moreover, the Ussing chamber experiments showed that electrogenic glucose transport in the jejunum was significantly enhanced after TAC administration. We also revealed transcription and expression changes in SGLT1 after TAC administration, which represents a promising therapeutic target for new-onset diabetes after transplantation.

We also found that food intake and body weight were not significantly affected by TAC intake. The possible reason for this may be that 2 weeks of TAC administration was not long enough to generate typical chronic symptoms of diabetes, including increased food intake and weight loss. However, the OGTT showed that blood glucose levels were elevated after TAC administration in low, medium, and high dose groups. A reasonable explanation for this phenomenon is likely impairment of glucose tolerance and/or increasing intestinal glucose absorptive capacity.

The effect of TAC on plasma insulins level remains controversial. Previous studies have found that plasma insulin levels after TAC administration decreased due to the impairment of islet β-cell function mediated through depleting islet β-cell amount, or reducing insulin synthesis or release [11]. However, Weiguo *et al*. found markedly elevated insulin resistance levels in TAC-treated recipients following kidney transplantation, and illustrated a positive correlation between TAC concentration and insulin resistance [24]. A 2-year follow-up cohort study on kidney transplant patients found that blood insulin levels in NODAT were significantly elevated and insulin resistance was the primary cause of TAC-induced NODAT [25]. In our work, serum insulin and blood glucose levels were both elevated in the medium and high groups, which suggests that insulin sensitivity was impaired by medium and high, but not low TAC doses, showing a dose dependent TAC effect on insulin sensitivity and induce insulin resistance. This finding is in accordance with earlier research in rats [26]. Recently, Pereira *et al*. have found that GLUT4 has been removed from the cell surface via an increased rate of endocytosis with therapeutic concentrations of TAC and glucose uptake was inhibited independent of insulin signaling, which is a novel mechanism for how TAC contributes to the development of insulin resistance and diabetes [27]. According to our results and previous published studies, we think that hyperglycemia initially occurred due to an increase in intestinal glucose absorption when TAC was administrated, and sustained hyperglycemia led to increased insulin secretion. The mice gradually became resistant to the effects of insulin as the receptors that bind to the hormone become less sensitive to insulin concentrations resulting in hyperinsulinemia and disturbances in insulin release [28]. With a reduced response to insulin, the β-cells secrete increasing amounts of insulin in response to the continued high blood glucose levels resulting in hyperinsulinemia, which is defined as a condition in which there are excess levels of insulin circulating in the blood than expected relative to the level of glucose [29]. Toxic effects of hyperglycemia [30], toxicity of TAC [11], and excessive secretion by β-cells due to insulin resistance, all contributed to impairment of β-cell function, which eventually lead to hypoinsulinemia.

Interestingly, by comparing intestinal morphology, we found the villus, crypt, and the villus surface, which represent the absorptive area, were not significantly changed in any experiment group after TAC administration. Moreover, combined with the OGTT result, suggests that blood glucose elevation in the experiment groups were not due to the enlargement of intestinal absorption area. Reasonably, the discordance between the unchanged intestinal morphology and increased intestinal glucose absorption is potentially instructive.

We focused our research on SGLT1, rather than other glucose transporters. SGLT1 is the dominating glucose transporter in the intestine [31], thus we estimated glucose absorptive capacity by the Ussing chamber experiments. We found that electrogenic glucose transport in low, medium, and high groups was significantly enhanced after TAC administration. Furthermore, this increase was not significantly different between the three groups, which indicates that the enhancement of glucose transportation is not dependent on TAC dose. Meanwhile, in our studies, glucose transport measured by the Ussing chamber was almost completely inhibited by LX-4211, an inhibitor of SGLT1, which further supported the role of SGLT1 in glucose transport in the intestine. Much of the research about the effect of immunosuppressants on intestine absorption over the last two decades have examined the effect of TAC on intestine glucose absorption, which produced conflicting results. Some studies found that TAC had no direct and immediate influence on rat intestinal glucose absorption in therapeutic concentrations [32, 33]. However, another study revealed glucose malabsorption in the jejunum after 6 weeks of TAC treatment and the effect revealed dose dependency [14]. Interestingly, one study observed that glucose absorption increased in the jejunum and decreased in the ileum of rats chronically administrated with TAC [34].

Consistent with our electrophysiological results, the protein abundance of SGLT1 in the intestine was higher in experimental versus control groups. Although SGLT1 plays a pivotal role in intestinal glucose absorption [31], we also measured the transcription and expression of other glucose transporters in the gut, such as GLUT2 and GLUT5. The mRNA and protein level of these transporters did not change after TAC administration, irrespective of dose. Thus, we believe that the enhancement of intestine glucose transport resulted from increased expression of the glucose transporter SGLT1.

Throughout the entire experiment, our research focus was on glucose absorption in the intestines to research the mechanism of the diabetogenic effect in mice. First, we applied electrophysiological technology, the Ussing chamber, which is an apparatus for measuring epithelial membrane properties, and can quantify transportation and barrier functions of living tissue, such as measuring the short-circuit current as an indicator of net ion transport taking place across an epithelium. Compared with other methods, the Ussing chamber is more consistent with physiological condition in vivo. Moreover, we could conveniently perform inhibition tests, which makes our results more credible. Finally, we used molecular biology tests to verify the increased expression of SGLT1.

In conclusion, our study showed enhanced glucose absorption in the jejunum by upregulated expression of SGLT1 induced by TAC. Moreover, administration of TAC also resulted in insulin resistance. Taking together, as the main executor of intestinal glucose absorption, SGLT1 represents a potential therapeutic target for new-onset diabetes after transplantation.

## Supporting Information

S1 File Detailed data of body weight, food intake, and random blood glucose levels. (XLSX)

S2 File Detailed information of TAC concentration of each mouse. (XLSX)

S3 File Glucose and insulin concentrations at each time point during the OGTT procedure. (XLSX)
